# Editorial: Cytochromes P450, their modulators and metabolites in cardiovascular function and disease

**DOI:** 10.3389/fphar.2024.1531166

**Published:** 2024-12-03

**Authors:** Adeniyi M. Adebesin, Richard Joseph Roman, William B. Campbell, John M. Seubert, Rheem A. Totah

**Affiliations:** ^1^ Department of Biochemistry, University of Texas Southwestern Medical Center, Dallas, TX, United States; ^2^ Department of Pharmacology and Toxicology, University of Mississippi Medical Center, Jackson, MS, United States; ^3^ Department of Pharmacology and Toxicology, Medical College of Wisconsin, Milwaukee, WI, United States; ^4^ Department of Pharmacology, Faculty of Pharmacy/Pharmaceutical Sciences, University of Alberta, Edmonton, AB, Canada; ^5^ Department of Medicinal Chemistry, University of Washington, Seattle, WA, United States

**Keywords:** cardiovascular diseases (CVD), cardiac hypertrophy, epoxyeicosatrienoic acid (EETs), hydroxyeicosatetraenoic acid (HETE), antihypertensive active components, cytochromes P450, soluble epoxide hydrolase (sEH)

Cardiovascular diseases (CVD) are major causes of morbidity and mortality despite the availability of a plethora of treatment options. This is partly due to our incomplete understanding of the complex and sometimes compensatory pathways involved in the initiation and progression of CVD. Therefore, advancements in the elucidation of CVD mechanisms are vital for new therapeutic drug development and improved patient care. With this perspective, this Research Topic (RT) on Cytochromes P450 (CYPs), Their Modulators and Metabolites in Cardiovascular Function and Disease comprises 1 review and 3 original research articles. This Research Topic of articles identifies emerging CYP pathways that contribute to CVD and highlights promising therapeutic approaches.

CYPs are heme-containing enzymes that mainly oxidize endogenous (e.g., fatty acids and steroids) and exogenous (e.g., drugs and xenobiotics) organic molecules to more hydrophilic metabolites. CYPs are differentially expressed and inducible in mammalian cells, and their expression and activity can regulate cellular and organ cross-talk and responses to diet and medications. A select number of CYPs are involved in the metabolism of polyunsaturated fatty acids (PUFAs), which is germane to cardiovascular homeostasis and disease. Therefore, a more complete understanding of the roles of these CYPs and their metabolites in controlling the cardiovascular system is of vital importance.

The review by Jiang et al. discusses the cardioprotective mechanisms of epoxyeicosatrienoic acids (EETs), epoxyeicosatetraenoic acids (EEQs), and epoxydocosapentaenoic acids (EDPs), which are CYP epoxygenase metabolites of arachidonic acid (AA), eicosapentaenoic acid (EPA), and docosahexaenoic acid (DHA), respectively. Research from various laboratories suggests that these metabolites are endothelium-derived hyperpolarization factors (EDHF) involved in blood vessel vasodilation and renal tubular salt reabsorption via actions on potassium, calcium, and sodium channels. Their proposed mechanisms of action include direct binding to ion channels and indirect modulation of ion channels via G-protein and protein kinase pathways. Therefore, EET mimetics and soluble epoxide hydrolase (sEH) inhibitors, which block epoxide hydrolysis of endogenous EETs, show significant blood pressure lowering effects in various hypertensive animal models. Other notable protective effects of EETs in CVD models include: 1) pro-angiogenesis and anti-apoptosis in myocardial infarction (MI) models, 2) anti-inflammation in atherosclerosis models, and 3) anti-oxidant effects and modulation of calcium homeostasis in heart failure models. The review also covers non-hydrolase functions of sEH in CVD, an often overlooked activity, as well as cell type-specific mechanisms of EETs and sEH inhibitors.

In the article by Baranowska et al., the combination of low-dose (10 mg/kg/day) of an orally-active, stable EET mimetic (EET-A) and a low dose (10 mg/kg/day) of AAA, a 20-hydroxyeicosatetraenoic acid (20-HETE) antagonist, was shown to provide superior blood pressure lowering effect than a higher dose (40 mg/kg/day) of EET-A alone in a spontaneously hypertensive rat model. 20-HETE is produced by the metabolism of arachidonic acid by the CYP4A and F subfamilies and is a vasoconstrictor in various vascular beds that contributes to the development of hypertension in SHR. These results indicate a prominent role for the 20-HETE/CYP4 pathway in the vascular dysfunction in this model and suggest a promising therapeutic strategy for the treatment of hypertension, which warrants additional investigation.

Helal et al. assessed the effects of individual (R/S)-enantiomers of 11-hydroxyeicosatetraenoic acid (11-HETE) on CYP1B1 activity and cellular hypertrophy in RL-14 cardiomyocyte cells. 11-HETE is an arachidonic acid metabolite produced by CYPs, cyclooxygenase (COX 1/2) enzymes, and non-enzymatic oxidative processes. Both enantiomers increased cardiomyocyte hypertrophy but only the 11(S)-enantiomer increased CYP1B1 activity. Interestingly, the 11(S)-enantiomer increased the mRNA and protein expression of both cardioprotective CYP2J and pro-inflammatory 20-HETE producing CYP4F2, while the 11(R)-enantiomer did not increase CYP2J levels. These results are consistent with previous work by the El-Kadi group on the differential effects of HETE enantiomers on CYP expression and activity that suggest the existence of distinct binding sites/receptors for each enantiomer. Other potential implications include: 1) a positive feed-back loop in the 11-HETE/CYP1B1 axis could exacerbate cardiac hypertrophy, 2) secondary metabolism of eicosanoids by 11-HETE induced CYPs might be relevant in cardiac hypertrophy, and 3) CYP expression and activity may encompass an equilibrium between anti-and pro-inflammatory eicosanoids, which potentially become skewed during oxidative stress or in diseased states. Strategies to restore the normal equilibrium between these isoforms might provide safer, more effective treatments for CVD.

Finally, using a multidisciplinary approach, Chu et al. elucidated the antihypertensive mechanism of action of tetrandrine, a bioactive alkaloid component of *Stephania tetrandra* S Moore root (Fang Ji), a traditional Chinese medicine used for decades for its diuretic and antihypertensive actions. They also demonstrated that chronic administration of tetrandrine (30 and 60 mg/Kg/day) to SHR was as effective as spironolactone (20 mg/Kg/day) in increasing sodium excretion and lowering blood pressure in spontaneously hypertensive rats. This work identifies tetrandrine as a diuretic that covalently binds to CYP11A1 in an unconventional way to inhibit aldosterone biosynthesis. The proposed mechanism should help researchers/clinicians understand its pharmacological effects and to explore future therapeutic applications, particularly for heart failure. Future work should investigate the inhibition of other CYPs by tetrandrine.

In summary, the articles in this RT contribute to our understanding of the roles of CYPs, their metabolites and modulators in CVD. The discoveries from these outstanding laboratories contained in this RT will stimulate further research on this Research Topic and spawn additional innovative ideas and therapies to improve cardiovascular health.

